# Meet the Editorial Board Member

**DOI:** 10.2174/1570159X2211240414123747

**Published:** 2024-04-14

**Authors:** J.H. Abraini

**Affiliations:** 1 University of Caen - Basse Normandie, Caen, France

   J.H. Abraini is the President of the International Group for High-Pressure Biology (IGHPB). He is a Professor of Neuroscience at the University of Caen Medical School (France) and at the Laval University Medical School, Department of Neuroscience and Psychiatry (QC, Canada). He has published many scientific publications and his fields of interest are Neuroprotection, Neurotransmission, Gamma-aminobutyric acid, neuropharmacological investigations and postsynaptic dopamine receptors.
